# Associations of demographic, socioeconomic, lifestyle factors and comorbidity with accelerometer-measured physical activity in adults with cardiovascular diseases

**DOI:** 10.1371/journal.pone.0352673

**Published:** 2026-07-14

**Authors:** Adina König, Maria Hagströmer, Frida Bergman, Maria Bäck, Isabel Drake, Henrik Johansson, Jenny Rossen, Philip von Rosen

**Affiliations:** 1 Division of Physiotherapy, Department of Neurobiology, Care Sciences and Society, Karolinska Institutet, Stockholm, Sweden; 2 Department of Health Promoting Science, Sophiahemmet University, Stockholm, Sweden; 3 Academic Primary Health Care Centre, Region Stockholm, Stockholm, Sweden; 4 Department of Public Health and Clinical Medicine, Umeå University, Umeå, Sweden; 5 Department of Health, Education and Technology, Division of Health, Medicine and Rehabilitation, Luleå University of Technology, Luleå, Sweden; 6 Department of Health, Medicine and Caring Sciences, Unit of Physiotherapy, Linköping University, Linköping, Sweden; 7 Department of Occupational Therapy and Physiotherapy, Sahlgrenska University Hospital, Gothenburg, Sweden; 8 Department of Molecular and Clinical Medicine, Institute of Medicine, Sahlgrenska Academy, University of Gothenburg, Gothenburg, Sweden; 9 Department of Clinical Sciences in Malmö, Diabetes and Cardiovascular Disease-Genetic Epidemiology, Lund University, Malmö, Sweden; 10 Department of Gastroenterology, Skåne University Hospital, Malmö, Sweden; 11 Department of Medical Sciences, Clinical Physiology, Uppsala University, Uppsala, Sweden; 12 Department of Women’s and Children’s Health, Physiotherapy, Uppsala University, Uppsala, Sweden; 13 Department of Medical Sciences, Respiratory-, Allergy- and Sleep Research, Uppsala University, Uppsala, Sweden; Srebrnjak Children’s Hospital, CROATIA

## Abstract

Cardiovascular diseases (CVDs) are a leading cause of morbidity and mortality, and physical activity (PA) is central to secondary prevention. However, factors associated with PA among individuals with CVDs remain poorly understood, with few studies using accelerometer-based methods. This cross-sectional study examined the associations between accelerometer-measured PA and demographic, socioeconomic, lifestyle factors, and comorbidity in a large Swedish cohort of individuals with CVDs. Participants aged between 50 and 64 years with a confirmed diagnosis of angina pectoris, myocardial infarction, atrial fibrillation and flutter, heart failure, or stroke were included. Moderate-to-vigorous physical activity was measured using hip-worn triaxial accelerometers over seven consecutive days and categorized into tertiles of low, medium and high PA level. Demographic, socioeconomic, lifestyle factors, and comorbidities were assessed through questionnaires and registry data. Associations with PA levels were analyzed using multiple ordinal logistic regression. A total of 1,484 participants (32% women; median age 60.2 years) were included. Being in the oldest age group was significantly associated with lower odds of having a higher PA level (OR = 0.59; 95% CI [0.44, 0.77]), as well as female sex (OR = 0.73; 95% CI [0.59, 0.91]), regular/occasional smoking (OR = 0.37; 95% CI [0.26, 0.52]), and having one (OR = 0.61; 95% CI [0.48, 0.77]) or more comorbidities (OR = 0.45; 95% CI [0.30, 0.68]). A healthy diet was significantly associated with higher PA levels (OR = 1.84; 95% CI [1.46, 2.31]). Financial strain, level of education and alcohol consumption had no association with PA. Using data from a representative sample of middle-aged adults, the findings indicate that engagement in PA is primarily associated with lifestyle factors, demographic characteristics, and comorbidity. These identified characteristics may inform future research aimed at CVD populations with low PA. Further research should investigate mediating and moderating pathways to clarify how these factors influence PA.

## Introduction

Cardiovascular diseases (CVDs) are the leading cause of the global disease burden, which is expected to rise due to population growth and ageing [1]. CVDs are the most prevalent non-communicable diseases and a major cause of mortality and morbidity [2], impacting individuals’ quality of life and economic growth [3]. CVDs encompass a range of conditions, with some of the most common being angina pectoris and myocardial infarction, as major clinical manifestations of coronary artery disease [2], as well as heart failure, stroke, and atrial fibrillation and flutter [1]. Physical activity (PA) is essential in secondary prevention of CVDs, as it helps prevent recurrent events [4] and is linked to lower rehospitalization [5] and risk of mortality [6]. However, factors associated with PA, ranging from demographic to health-related characteristics, remain underexplored in individuals with CVDs [7]. In this context, device-measured PA should be preferred when investigating associations with PA, as it provides a more precise quantification of PA compared to self-reported PA by limiting recall and response bias [8], although evidence from such studies remains limited.

Correlates of PA have been examined in the general adult population, with demographic factors such as age and sex among the most commonly studied [9,10]. Some studies report an inverse relationship between age and PA [11–13] and a positive association with being male [11,12], while other findings regarding sex are inconclusive [13–15]. In populations with CVDs, an inverse relationship of age and PA was reported in systematic reviews, where PA was assessed both through self-report and device-based measures in patients with heart failure [16] and post-stroke [17]. Socioeconomic factors are also investigated as correlates of PA, including education and income, which are both determining factors for individuals regarding the capacity and opportunity to make informed health decisions [18]. In adults from the general population, the majority of studies report a positive association between socioeconomic factors and predominantly self-reported PA [9,10]. However, Bauman and colleagues [9] point out that this positive association primarily applies to low- and middle-income countries, whereas it is inconsistent or even inverse in high-income countries. An American study with nearly 17,000 self-identified post-stroke patients analyzing the relationship between self-reported PA and socioeconomic factors, found that higher education was associated with a higher PA level [19].

Furthermore, PA is related to lifestyle factors such as smoking, alcohol consumption and dietary habits. A systematic review including mixed populations found that a “healthy lifestyle” cluster commonly includes being physically active, alongside non-smoking and adherence to dietary and alcohol guidelines [20]. These lifestyle factors are key modifiable risk factors in the secondary prevention of CVDs, as their improvement can considerably reduce adverse health outcomes in people with CVDs [21]. Another important factor associated with PA is multimorbidity, which is the co-occurrence of several, often chronic diseases [22]. Multimorbidity is likely to occur in people with CVDs [23] and affects both mental and especially physical health [24]. When chronic diseases occur alongside a CVD, they are also referred to as comorbidities [23]. Recent cross-sectional studies suggest that having multiple chronic diseases is associated with less time spent in PA [25,26].

PA is associated with multiple factors that have predominantly been explored in general or mixed populations. Research focusing on individuals with CVDs is limited, often constrained by small sample sizes or reliance on self-reported PA measures. A better understanding of factors associated with PA within CVD populations is essential for identifying distinguishing characteristics between individuals with low and high PA levels. Such knowledge can inform the development of more targeted interventions to promote PA and hence support secondary prevention in people with CVDs. Therefore, this study aims to examine the associations between accelerometer-measured PA and demographic, socioeconomic, lifestyle factors, as well as comorbidity in individuals diagnosed with CVDs.

## Materials and methods

### Study design, recruitment and data collection

This is a cross-sectional study using data from the Swedish CArdioPulmonary BioImage Study (SCAPIS) cohort. The overall aim of SCAPIS is to better understand the underlying mechanisms of cardiopulmonary and metabolic diseases [27]. SCAPIS was conducted between 04/11/2013 and 04/10/2018 and included individuals who were randomly selected from the general population [26]. The inclusion criteria were that participants needed to be between 50 and 64 years old and understand written and spoken Swedish to give informed consent [27]. Data collection was performed at six university hospital sites across Sweden. At the first visit, participants received an accelerometer to measure PA and filled out an extensive questionnaire, which included questions about lifestyle, education and financial situation [26]. Information on age and sex was retrieved from national registry data. SCAPIS invited a total of 59,909 participants, out of which 30,154 participated [26].

The present study was approved by the Swedish Ethical Review Authority (Dnr 2022-00591-02 and 2021-05639-01) and data were accessed for research purposes on 02/03/2022.

### Cardiovascular diseases (CVDs)

The CVD diagnoses were retrieved from the inpatient and specialized outpatient registry of the Swedish National Board of Health and Welfare using codes from the International Classification of Diseases (ICD) version 10. Participants from the SCAPIS cohort with the following CVDs and respective ICD-10 codes were included:

- Angina pectoris: I20
- Myocardial infarction: I21, I22, I25.2
- Atrial fibrillation and flutter: I48
- Heart failure: I50
- Stroke: I61, I63, I64

To minimize the inclusion of possible resolved diseases, only participants with diagnoses after the year 2000 were included. Further, participants were included if the date of the first visit for data collection came after their date of diagnosis, to ensure that participants already had the diagnosis when enrolling in SCAPIS. A flowchart presenting participant inclusion is provided in Supporting information (S1 Fig).

### Physical activity

PA was measured using either one of the following triaxial accelerometer models: ActiGraph GT3X + , wGT3X + , wGT3X-BT or ActiGraph LCC, all manufactured by ActiGraph LLC, Pensacola, FL, USA [26]. Participants were asked to wear the accelerometer on the right hip for seven consecutive days during waking hours, except during water-based activities [26]. Only participants with valid wear time were included, which was defined as having a minimum of 600 minutes of daily wear time on at least four days [28]. In total, 27,890 participants provided valid accelerometer data [26]. The software ActiLife (v.6.13.3) was used to initialize the accelerometers and to download and process the collected raw data [26]. Accelerometers recorded triaxial activity at a sampling rate of 30 Hz. The raw data were combined into a vector magnitude, extracted as 60-second epochs using a low-frequency extension filter, and converted into counts per minute (cpm). PA was defined as time spent in moderate-to-vigorous physical activity (MVPA), where MVPA was defined as ≥ 2,690 counts per minute [29].

### Demographic factors

Information about participants’ age and sex was retrieved from the Swedish population register. Participants were assigned to one of three age categories: 50–54 years old, 55–59 years old or 60–65 years old. Sex comprised being either male or female.

### Socioeconomic factors

Socioeconomic factors, obtained via questionnaires, included information on financial strain and the highest completed level of education. Having financial strain was determined by answering “Yes” or “No” to the question, “If you were to suddenly end up in a situation where you had to raise SEK 20,000 (≈ EUR 1,800) in one week, would you be able to do it?” Regarding the level of education, participants were divided into three categories: “Primary school or lower”, “Secondary school” and “University degree”.

### Lifestyle factors

Lifestyle factors included participants’ alcohol consumption, their smoking status, and adherence to dietary guidelines, which were assessed through questionnaires. The alcohol consumption was assessed using the Alcohol Use Disorders Identification Test (AUDIT) [30] with a slight modification: Instead of the original three answer options (No; Yes, but not in the last year; Yes, during the last year) for the last two questions, SCAPIS had two answer options (Yes; No). The questionnaire asks 10 questions about personal alcohol consumption and its consequences, with a maximum possible score of 40 points. Based on the final score, participants were categorized as having “No harmful alcohol use” (< 8 points) or “Harmful alcohol use” (≥ 8 points). The AUDIT is a valid and reliable tool to assess harmful drinking in different populations [31]. Regarding the smoking status, participants had four response alternatives to the question “Do you smoke?” (No, I never smoked; No, I stopped smoking; Yes, I smoke occasionally; Yes, I smoke regularly). Participants were assigned to one of the three categories: “Regular/occasional smoker”, “Former smoker” and “Never smoked”. Adherence to dietary guidelines was assessed using the Swedish dietary guideline index (SweDGI) [32], which reflects adherence to dietary guidelines issued by the National Food Agency [33]. The SweDGI covers intake of encouraged foods (fruits, vegetables, legumes, nuts and seeds, whole grains, oil-dressing and fish and shellfish) and foods to limit (red and processed meat, high-fat dairy products, added sugar, alcohol contained in food and salt). Each component is scored from 0 (very poor adherence) to 4 (very good adherence), forming a total score ranging from 0 to 48, with higher scores indicating better adherence to dietary guidelines. Participants were initially categorized into five groups based on their cumulative score: ≤ 18 (group 1), 19–22 (group 2), 23–25 (group 3), 26–29 (group 4), and ≥30 points (group 5). For the present study, these categories were further collapsed into three groups: “Unhealthy diet” (groups 1 & 2), “Moderately healthy diet” (group 3) and “Healthy diet” (groups 4 & 5). The SweDGI uses dietary intake data from the validated MiniMeal-Q food frequency questionnaire [34].

### Comorbidity

A Swedish-adapted version of the Charlson Comorbidity Index (CCI) was used to determine comorbidity [35]. This version by Ludvigsson and colleagues was modified for the present study in the following way: CVDs listed in the Swedish CCI, which included myocardial infarction, congestive heart failure, and cerebrovascular disease, were excluded as they are already registered as CVDs. The final modified CCI comprised 16 comorbidities, including diabetes, chronic obstructive pulmonary disease (COPD), liver disease and malignancies. Each comorbidity counted as one point, meaning the maximum possible score was 16. Participants were divided into the three comorbidity categories of “No comorbidity”, “One comorbidity” and “Two or more comorbidities”. The CCI was originally developed to predict the risk of mortality [36] and is now also used to adjust for comorbidity burden in epidemiological studies [35]. The content validity of the Swedish-adapted CCI is strengthened by harmonization of ICD codes across versions for accurate disease representation and by expert review of all included codes [35].

### Statistical analysis

Out of the included participants (n = 1,484), 8% had missing values on at least one of the socioeconomic or lifestyle factors. Missing data were present in education (3%), financial strain (5%), smoking status (4%) and alcohol consumption (4%). Missing values were imputed using multiple imputation (MI). Five datasets were generated using multivariate imputation by chained equation in R (mice package) [37]. MI is a technique for handling missing data by creating different plausible datasets, which reflect the uncertainty of the missing data [38].

Multicollinearity was assessed using the variance inflation factor (VIF). Most variables indicated a VIF of < 3, while the level of education (VIF = 6.63) and age (VIF = 4.78) showed an elevated VIF. Following O’Brien and considering our large sample size and the theoretical relevance of these variables, we retained the variables with elevated VIF values [39]. Based on time spent in MVPA, participants were split into tertiles of low, medium and high PA level. Multiple ordinal logistic regression was applied to investigate the associations between the PA level (dependent variable) and demographic, socioeconomic, lifestyle factors and comorbidity (independent variables). The independent variables were selected based on existing literature demonstrating their relevance to key characteristics among people with CVDs. Multiple ordinal logistic regression was conducted separately on each imputed dataset, and results were pooled using Rubin’s rules to obtain the estimates. The regression coefficients were exponentiated to calculate the odds ratios. To assess the robustness of the results based on imputed data, a complete-case analysis was performed using observations with no missing data (n = 1,373). The complete-case analysis showed results comparable to those from the analysis using imputed data. The presented results are based on imputed data.

Multiple ordinal logistic regression and assumption testing were performed using the ordinal package in R [40]. The proportional odds assumption was tested using the nominal test and was conducted on each imputed dataset, where the assumption was consistently met (all p-values > 0.05). Data analysis was conducted using R version 4.5.3 (R Core Team, 2026) with a level of statistical significance set at 0.05.

## Results

The final sample included 1,484 participants (32% women) with a median age of 60.2 years, and their characteristics are presented in Table 1, as well as the characteristics for each PA tertile. Across the whole sample, the median time spent in MVPA per day was 45 minutes. The majority completed secondary school (48%) and had no financial strain (85%). Around half of the participants were either regular/occasional smokers (12%) or former smokers (42%), 8% of participants had harmful alcohol consumption, and around half of the participants had an unhealthy diet (51%). The majority had no comorbidity (71%). The median time between the diagnosis and enrolling in SCAPIS was 5.8 years.

**Table 1:** Characteristics of the whole study sample and the PA tertiles showing the frequencies and percentages (in parentheses), except for moderate-to-vigorous physical activity; Missing values were present for education, financial strain, smoking status and alcohol consumption.

Characteristic	Whole sample(n = 1,484)	Low PA tertile (n = 517)	Medium PA tertile(n = 480)	High PA tertile(n = 487)
Age (years)				
Age group 50–54	244 (16)	64 (12)	82 (17)	98 (20)
Age group 55–59	482 (33)	158 (31)	156 (33)	168 (35)
Age group 60–65	758 (51)	295 (57)	242 (50)	221 (45)
Sex				
Women	478 (32)	180 (35)	158 (33)	140 (29)
Men	1,006 (68)	337 (65)	322 (67)	347 (71)
Education^c^				
Primary school or lower	207 (14)	79 (15)	62 (13)	66 (14)
Secondary school	714 (48)	274 (53)	232 (48)	208 (43)
University degree	515 (35)	143 (28)	173 (36)	199 (41)
Financial strain^a,c^				
Yes	1,255 (85)	419 (81)	415 (86)	421 (86)
No	148 (10)	59 (11)	46 (10)	43 (9)
Smoking status^c^				
Never smoked	621 (42)	168 (32)	212 (44)	241 (49)
Former smoker	623 (42)	225 (44)	196 (41)	202 (42)
Regular/occasional smoker	182 (12)	98 (19)	57 (12)	27 (6)
Alcohol consumption^c^				
Not harmful	1,305 (88)	448 (87)	430 (90)	427 (88)
Harmful	120 (8)	43 (8)	36 (7)	41 (8)
Diet				
Unhealthy	752 (51)	300 (58)	248 (52)	204 (42)
Moderately healthy	242 (16)	89 (17)	77 (16)	76 (16)
Healthy	490 (33)	128 (25)	155 (32)	207 (42)
Comorbidity				
No comorbidity	1,051 (71)	312 (60)	357 (74)	382 (79)
One comorbidity	333 (22)	149 (29)	100 (21)	84 (17)
Two or more comorbidities	100 (7)	56 (11)	23 (5)	21 (4)
MVPA (min/day)^b^	45 (29-65)	23 (16 –29)	45 (40 –51)	77 (65-95)

Abbreviations: Physical activity (PA), Moderate-to-vigorous physical activity (MVPA)

^a^Answering the question “If you were to suddenly end up in a situation where you had to raise SEK 20,000 (≈ EUR 1,800) in one week, would you be able to do it?”

^b^Median and interquartile range

^c^Contains missing values

The associations between PA levels and demographic, socioeconomic, lifestyle factors and comorbidity are shown in Table 2. Being in the oldest age group was significantly associated with lower odds of having a higher PA level compared to the youngest age group (OR = 0.59; 95% CI [0.44, 0.77]). Being female was significantly associated with lower odds of having a higher PA level compared to being male (OR = 0.73; 95% CI [0.59, 0.91]). Being a regular/occasional smoker was significantly associated with lower odds of having a higher PA level compared to non-smokers (OR = 0.37; 95% CI [0.26, 0.52]). Having a healthy diet was significantly associated with higher odds of having a higher PA level compared to having an unhealthy diet (OR = 1.84; 95% CI [1.46, 2.31]). Having one comorbidity (OR = 0.61; 95% CI [0.48, 0.77]) or two or more comorbidities (OR = 0.45; 95% CI [0.30, 0.68]) was significantly associated with lower odds of being in a medium or high PA category compared to having no comorbidities. Financial strain, level of education and alcohol consumption had no significant association with the PA level.

**Table 2:** Odds ratios from the multiple ordinal logistic regression with physical activity level (low, medium, high) as the dependent variable. The odds ratios indicate the likelihood of being in a higher physical activity tertile compared with a lower tertile for each independent variable relative to its reference category (Ref). (Results are pooled across five imputations).

Independent variable	Odds Ratio (95% CI)	P-Value	Std. Error
Age group 50–54 years (Ref) Age group 55–59 years Age group 60–65 years	0.77 (0.58–1.03) 0.59 (0.44–0.77)	0.080 <0.001	0.15 0.14
Sex (male) (Ref) Sex (female)	0.73 (0.59–0.91)	0.010	0.11
Primary school or lower (Ref) Secondary school University degree	0.74 (0.55–0.99) 0.99 (0.72–1.38)	0.050 0.970	0.15 0.17
Financial strain (Yes) (Ref) Financial strain (No)	1.09 (0.78–1.53)	0.600	0.17
Never smoked (Ref) Former smoker Regular/occasional smoker	0.82 (0.66–1.02) 0.37 (0.26–0.52)	0.070 <0.001	0.11 0.18
No harmful alcohol consumption (Ref) Harmful alcohol consumption	1.30 (0.90–1.87)	0.160	0.19
Unhealthy diet (Ref) Moderately healthy diet Healthy diet	1.24 (0.94–1.63) 1.84 (1.46–2.31)	0.130 <0.001	0.14 0.12
No comorbidity (Ref) One comorbidity Two or more comorbidities	0.61 (0.48–0.77) 0.45 (0.30–0.68)	<0.001 <0.001	0.12 0.21

Based on the statistically significant results of the multiple ordinal logistic model, the predictive probabilities showed that participants in the oldest age group had a 44.9% probability of having a low PA level and a 24.4% probability of having a high PA level. Women were more likely to have a low (42.3%) rather than a high (26.5%) PA level. Regular/occasional smokers were more likely to have a low (52.4%) than a high (18.6%) PA level. Participants with a healthy diet were more likely to have a high PA level (36.1%) compared with a low PA level (32%). Participants with one comorbidity were more likely to have a low (40.2%) rather than a high (27.8%) PA level, and those with two or more comorbidities were more likely to have a low (47.0%) than a high (22.6%) PA level.

## Discussion

This cross-sectional study investigated the associations between accelerometer-measured PA and demographic, socioeconomic, lifestyle factors and comorbidity in individuals diagnosed with CVDs from a large Swedish cohort. Lower PA levels were significantly associated with older age, female sex, regular or occasional smoking, and having one or more comorbidities. Conversely, a healthy diet was significantly associated with higher PA levels.

The most notable findings of our study are that older age (≥ 60 years), female sex, regular or occasional smoking, and having two or more comorbidities were among the factors associated with the highest probabilities of a low PA level in people with CVDs. This indicates that demographic, lifestyle factors and comorbidities are strongly associated with PA levels in this population. The strong negative association with smoking and the positive association with a healthy diet highlight the clustering of lifestyle behaviors, where individuals engaging in one healthy behavior are more likely to engage in others [20]. Interestingly, harmful alcohol consumption showed no association with PA. This lack of association may partly reflect the small proportion of participants reporting harmful alcohol use (8%). Additionally, the use of a modified AUDIT version, compared to the original instrument, may have increased the likelihood of higher scores, as only two response options were available for the last two items, potentially leading to misclassification and an attenuated association with PA. The relationship between comorbidities and PA in our study is in line with results from previous studies, including the whole SCAPIS cohort [25,26], as well as studies including participants with coronary artery disease [41,42]. Stewart and colleagues report that study participants with poorer general health, cardiac and non-cardiac comorbidities were likely to be less physically active [42]. However, in Huang and colleagues’ study [41], the negative association between comorbidities and PA only applied to men. The negative association that we observed between PA and age confirms findings from studies including adults from the general population [9,10] as well as people with CVDs [16,17]. Age has a changing association with PA across the lifespan, whereby PA levels may fluctuate during middle adulthood before becoming lower in older age [43].

In this study, female sex was significantly associated with lower PA levels. This aligns with findings from general population studies, where women report lower PA compared to men [11,12]. Findings in CVD populations are inconsistent, with no significant associations between sex and PA [16] or weak correlations [17]. Viewing the whole SCAPIS sample, Ekblom-Bak and colleagues found that men engage in more vigorous PA than women, whereas women engage in more light PA [26], suggesting that the intensity of PA plays a role when comparing between sexes.

The level of education and financial strain showed no association with PA. However, the association between completion of secondary school and PA was close to the threshold for statistical significance, which should be interpreted with caution. The limited socioeconomic variability in the sample, where the majority had completed at least secondary school (83%) and reported no financial strain (85%), as well as the elevated VIF for education, may have affected the precision of the estimates. Arora and colleagues found that higher education was associated with a higher PA level in participants with stroke [19], which was based on bivariate analyses and did not account for confounding factors. Our multivariable approach suggests that education may contribute to the PA level, but its role appears limited when other factors are adjusted for.

A strength of this study is the use of data from the large population cohort SCAPIS, which includes accelerometer-measured PA from 27,890 participants [26]. Additionally, SCAPIS data are linked to national registry data, which enables the retrieval of the CVD diagnoses. Compared with the general Swedish population, SCAPIS participants show a significant but small difference in education (higher education in the SCAPIS cohort) and proportion of never-smokers (lower in the SCAPIS cohort) [26], and the alcohol consumption appears to be higher in the SCAPIS cohort [44]. Using multiple ordinal logistic regression enabled us to investigate associations between several factors and PA, providing a more realistic picture compared to bivariate analyses. MI served as an adequate method to retain observations with valid accelerometer data and missing values in the independent variables. Measuring PA using accelerometers reduces recall and response bias, compared to self-reported measures [8]. Hip-worn accelerometers can measure total body movement [45], but are limited in capturing activities, such as cycling, water-based and upper-body activities [46]. We used MVPA as an overall measure of PA and divided participants into tertiles to distinguish between PA levels. A limitation of this approach is that individuals near the tertile cut points may fall into different groups despite having similar MVPA values, reducing variability and statistical power. Nevertheless, we consider this approach practical as it facilitates interpretation and comparison across PA levels. We are aware that sedentary behavior is an important movement behavior alongside PA. However, the focus of this study was PA as a single movement behavior and its relationship with other relevant variables. Future research could apply compositional analysis, considering a combined distribution of sedentary behavior and PA, to provide a more comprehensive understanding of movement behaviors in CVD populations. Importantly, the associations in this study are correlational and not to be interpreted as causal.

## Conclusions

This study addressed the gap in PA correlates in individuals with CVDs by using accelerometer-measured PA from a large Swedish cohort. Lower PA was significantly associated with older age, female sex, regular or occasional smokers, and comorbidities, while a healthy diet was significantly associated with higher PA levels. Financial strain, level of education and alcohol consumption showed no association. These findings highlight characteristics linked to PA levels, which may inform the design of future research aimed at CVD populations with low activity profiles in need of PA promotion. Further research should investigate potential mediating and moderating pathways to clarify how these factors interact and influence PA levels.

## Supporting information

S1 Fig Flowchart of participant inclusion. (DOCX)

S2 File STROBE checklist. (DOCX)
