# Supplementary material for: Associations of demographic, socioeconomic, lifestyle factors and comorbidity with accelerometer-measured physical activity in adults with cardiovascular diseases
Source: PLoS One. 2026 Jul 14;21(7):e0352673. doi: 10.1371/journal.pone.0352673 (PMC13367697; doi:10.1371/journal.pone.0352673)
Supplement: S1 Fig — (DOCX) [file pone.0352673.s001.docx]

Gave informed consent to participate
**(n = 30,154)**

Participants with valid accelerometer wear time **(n = 27,890)**

Participants with CVD diagnoses after the year 2000 **(n = 2,141)**

Exclusion of participant with non-valid accelerometer wear time
**(n = 2,264)**

Exclusion of participants with date of diagnoses after inclusion in SCAPIS
**(n = 657)**

Participants included in the present study **(n = 1,484)**

Exclusion of participants with no CVD diagnosis**)** and CVD diagnoses before the year 2000 **(n = 25,749)**

Invited to participate in SCAPIS
 **(n = 59,909)**

Declined or no response
**(n = 29,755)**

**S1 fig**: Flowchart of participant inclusion.
