# Supplementary material for: Associations of demographic, socioeconomic, lifestyle factors and comorbidity with accelerometer-measured physical activity in adults with cardiovascular diseases
Source: PLoS One. 2026 Jul 14;21(7):e0352673. doi: 10.1371/journal.pone.0352673 (PMC13367697; doi:10.1371/journal.pone.0352673)
Supplement: S2 File — (DOCX) [file pone.0352673.s002.docx]

STROBE Statement—checklist of items that should be included in reports of observational studies

|  | Item No. | Recommendation | Page  No. | Relevant text from manuscript |
| --- | --- | --- | --- | --- |
| **Title and abstract** | 1 | (*a*) Indicate the study’s design with a commonly used term in the title or the abstract |  | Cross-sectional (abstract). |
|  |  | (*b*) Provide in the abstract an informative and balanced summary of what was done and what was found |  |  |
| Introduction | | | |  |
| Background/rationale | 2 | Explain the scientific background and rationale for the investigation being reported | 1-2 | The rising cardiovascular disease burden and factors associated with physical activity (PA) are underexplored in individuals with cardiovascular diseases (CVDs). |
| Objectives | 3 | State specific objectives, including any prespecified hypotheses | 2 | Examine associations between accelerometer-measured PA and demographic, socioeconomic, lifestyle factors, and comorbidity in individuals with CVDs. |
| Methods | | | |  |
| Study design | 4 | Present key elements of study design early in the paper | 2 | We conducted a cross-sectional study using data from the SCAPIS cohort. |
| Setting | 5 | Describe the setting, locations, and relevant dates, including periods of recruitment, exposure, follow-up, and data collection | 2 | Data was collected at six Swedish hospital sites between 2013 and 2018. |
| Participants | 6 | (*a*) *Cohort study*—Give the eligibility criteria, and the sources and methods of selection of participants. Describe methods of follow-up  *Case-control study*—Give the eligibility criteria, and the sources and methods of case ascertainment and control selection. Give the rationale for the choice of cases and controls  *Cross-sectional study*—Give the eligibility criteria, and the sources and methods of selection of participants | 2 | Participants needed to be between 50 and 64 years old and understand written and spoken Swedish to give informed consent. |
|  |  | (*b*) *Cohort study*—For matched studies, give matching criteria and number of exposed and unexposed  *Case-control study*—For matched studies, give matching criteria and the number of controls per case |  |  |
| Variables | 7 | Clearly define all outcomes, exposures, predictors, potential confounders, and effect modifiers. Give diagnostic criteria, if applicable | 2-4 | Dependent variable: PA level; independent variables: age, sex, education level, financial strain, smoking, diet, alcohol consumption, comorbidity |
| Data sources/ measurement | 8* | For each variable of interest, give sources of data and details of methods of assessment (measurement). Describe comparability of assessment methods if there is more than one group | 2-4 | PA was measured using triaxial accelerometers. Age, sex, education level, financial strain, smoking, diet, alcohol consumption, were assessed using questionnaires, and comorbidity through a modified Charlson Comorbidity Index. Full measurements are presented in the methods section. |
| Bias | 9 | Describe any efforts to address potential sources of bias | 8 | To limit recall and response bias in the PA measurements, accelerometers were used. |
| Study size | 10 | Explain how the study size was arrived at | 2 | A flowchart (Supplementary Figure 1) presenting participant inclusion can be found in Additional file 1. |

Continued on next page

| Quantitative variables | 11 | Explain how quantitative variables were handled in the analyses. If applicable, describe which groupings were chosen and why | 2-4 | Quantitative variables were categorised to improve model interpretability, and the specific cut-off points are described in the methods section. |
| --- | --- | --- | --- | --- |
| Statistical methods | 12 | (*a*) Describe all statistical methods, including those used to control for confounding | 4 | Multiple ordinal logistic regression was applied to investigate the associations between the PA level and demographic, socioeconomic, lifestyle factors and comorbidity. Multicollinearity was assessed using the variance inflation factor. |
|  |  | (*b*) Describe any methods used to examine subgroups and interactions |  |  |
|  |  | (*c*) Explain how missing data were addressed | 4 | Missing data were imputed using multiple imputation. |
|  |  | (*d*) *Cohort study*—If applicable, explain how loss to follow-up was addressed  *Case-control study*—If applicable, explain how matching of cases and controls was addressed  *Cross-sectional study*—If applicable, describe analytical methods taking account of sampling strategy |  |  |
|  |  | (*e*) Describe any sensitivity analyses | 4 | A complete case analysis was performed using observations with no missing data to assess the robustness of the results based on imputed data. |
| Results | | | | |
| Participants | 13* | (a) Report numbers of individuals at each stage of study—eg numbers potentially eligible, examined for eligibility, confirmed eligible, included in the study, completing follow-up, and analysed | 5 | The final sample included 1.484 participants. |
|  |  | (b) Give reasons for non-participation at each stage |  |  |
|  |  | (c) Consider use of a flow diagram |  |  |
| Descriptive data | 14* | (a) Give characteristics of study participants (eg demographic, clinical, social) and information on exposures and potential confounders | 6 | Table 2 presents the characteristics of study participants. |
|  |  | (b) Indicate number of participants with missing data for each variable of interest | 4 | Missing data were present in education (3%), financial strain (5%), smoking status (4%) and alcohol consumption (4%). |
|  |  | (c) *Cohort study*—Summarise follow-up time (eg, average and total amount) |  |  |
| Outcome data | 15* | *Cohort study*—Report numbers of outcome events or summary measures over time |  |  |
|  |  | *Case-control study—*Report numbers in each exposure category, or summary measures of exposure |  |  |
|  |  | *Cross-sectional study—*Report numbers of outcome events or summary measures | 6 | The respective number of participants for each PA level are n=517 (low), n=480 (medium) n=487 (high). All summary measures are reported in the results section in Table 1. |
| Main results | 16 | (*a*) Give unadjusted estimates and, if applicable, confounder-adjusted estimates and their precision (eg, 95% confidence interval). Make clear which confounders were adjusted for and why they were included |  | No unadjusted estimates are reported. |
|  |  | (*b*) Report category boundaries when continuous variables were categorized | 3-4 | Based on time spent in moderate-to-vigorous physical activity (MVPA), participants were split into tertiles. Based on participants’ age, they were assigned to one of three age categories: 50-54 years old, 55-59 years old or 60-65 years old. |
|  |  | (*c*) If relevant, consider translating estimates of relative risk into absolute risk for a meaningful time period |  |  |

Continued on next page

| Other analyses | 17 | Report other analyses done—eg analyses of subgroups and interactions, and sensitivity analyses |  |  |
| --- | --- | --- | --- | --- |
| Discussion | | | | |
| Key results | 18 | Summarise key results with reference to study objectives | 7 | Older age, female sex, regular or occasional smokers, and having one or more comorbidities were significantly associated with lower PA levels, and a healthy diet was significantly associated with higher PA levels. |
| Limitations | 19 | Discuss limitations of the study, taking into account sources of potential bias or imprecision. Discuss both direction and magnitude of any potential bias | 8 | A limitation of splitting the sample into PA tertiles is that individuals near the tertile cut points may fall into different groups despite having similar MVPA values, reducing variability and statistical power. |
| Interpretation | 20 | Give a cautious overall interpretation of results considering objectives, limitations, multiplicity of analyses, results from similar studies, and other relevant evidence | 7-9 | Demographic, lifestyle factors and comorbidities, rather than socioeconomic characteristics, are strongly associated with PA levels in this CVD population. |
| Generalisability | 21 | Discuss the generalisability (external validity) of the study results | 8 | SCAPIS participants show higher education compared with the general Swedish population, a lower proportion of never-smokers, and higher alcohol consumption. |
| Other information | |  | | |
| Funding | 22 | Give the source of funding and the role of the funders for the present study and, if applicable, for the original study on which the present article is based | 12 |  |

*Give information separately for cases and controls in case-control studies and, if applicable, for exposed and unexposed groups in cohort and cross-sectional studies.

**Note:** An Explanation and Elaboration article discusses each checklist item and gives methodological background and published examples of transparent reporting. The STROBE checklist is best used in conjunction with this article (freely available on the Web sites of PLoS Medicine at http://www.plosmedicine.org/, Annals of Internal Medicine at http://www.annals.org/, and Epidemiology at http://www.epidem.com/). Information on the STROBE Initiative is available at www.strobe-statement.org.
